# Implementation of ERAS Protocols: In Theory and Practice

**DOI:** 10.4274/TJAR.2024.241723

**Published:** 2024-10-30

**Authors:** Menekşe Özçelik

**Affiliations:** 1 Ankara University Faculty of Medicine, Cebeci Hospital, Clinic of Anaesthesiology and Reanimation, Ankara, Turkey

**Keywords:** Enhanced recovery after surgery, implementation science, practice guideline, surgery

## Abstract

The enhanced recovery after surgery (ERAS) pathway is a perioperative care pathway intended to facilitate early recovery and minimize hospital stays among patients undergoing major surgery. Critical factors for successful ERAS implementation, which may vary depending on care processes, include a multidisciplinary team, organizational commitment to change, and a real-time system for compliance and outcome audits. As most clinicians and health organizations can attest, incorporating and implementing new evidence-based practice changes almost always involves overcoming systemic challenges and obstacles. The same holds true for ERAS programs. The main barriers to ERAS protocol implementation have been resistance to change, lack of time and resources, and inadequate communication and coordination among departments. According to evidence-based ERAS guidelines, the best way to efficiently implement all recommendations into practice is to discover. Implementation science aims to identify and address care gaps, support change in practice, and enhance healthcare quality. Implementation research should also build a robust and generalizable evidence base to inform implementation practice. Most implementation investigations focus on one of two approaches to achieving change. Implementation can progress through top-down or bottom-up processes depending on factors such as national policies, organizational properties, or the implementation culture of society, especially for health issues. Although the ERAS guidelines are based on evidence-based knowledge, only a limited number of health centers around the world have officially been able to implement them. The purpose of this review is to analyze the implementation of the ERAS pathways in theory and practice in Turkey, considering the absence of an ERAS-qualified center in Turkey.

**Main Points**

• Enhanced recovery after surgery (ERAS) is a patient-centered, evidence-based approach to improve perioperative care that involves col­laboration among different healthcare disciplines.

• There has been a remarkable surge in the adoption of ERAS protocols worldwide.

• Implementation science involves integrating evidence-based practices, interventions, and policies into routine healthcare.

• Every healthcare system, hospital, and organization may require distinct implementation strategies.

## Introduction

Enhanced recovery after surgery (ERAS) is a protocol-based pathway focusing on every step of perioperative care. The main fostering power of this concept was the realization that unimodal interventions did not address perioperative morbidity, which has a multifaceted genesis. Kehlet and Wilmore, the “father” of the ERAS concept, defined the essential mechanism of postoperative complications as the degree of pathophysiological stress response to surgery and subsequent organ dysfunction.^1^ Accordingly, to decrease postoperative morbidity, the stress response to surgery should be reduced. Advances in this era have improved patient outcomes, including reduced postoperative length of stay, significant cost savings, and increased patient satisfaction for those undergoing both open and laparoscopic colorectal surgery.^2^ In this context, multidimensional, multimodal, multidisciplinary, protocolized perioperative care bundles called ERAS protocols have been developed. Since the turn of the millennium, there has been a remarkable surge in the adoption and dissemination of protocols consisting of evidence-based interventions.

The ERAS pathways have been particularly pronounced in developed countries, indicating a significant shift in perioperative care practices. The concept has been accepted worldwide and continues to grow for almost every surgical specialty because of positive studies favoring ERAS protocols. However, despite such numerous clinical studies, there still needs to be more debate on whether ERAS implementation has the success it deserves. In this context, establishing the scientific and clinical benefits of ERAS protocols cannot guarantee their application in everyday clinical practice.

Advances in medical research have greatly extended human lifespans over the past century. A maximum of 50% of these medical research has been incorporated into routine use. Moreover, implementing an innovative approach into routine clinical practice usually takes 17-20 years.^3^ Considering that the first ERAS recommendation paper was published for colon surgery in 2005, it was expected that ERAS protocols would take their place among routinely applied protocols between 2022 and 2025.^4^ However, this differs from today’s reality, especially in developing countries. The issue is why ERAS guidelines are still not used in routine practice, even though the time frame mentioned in implementation science has passed.

This review investigates the difficulties in implementing ERAS protocols and their implementation into routine daily practice in Turkey. This study also aimed to identify appropriate strategies to overcome the main problems by considering the rules of implementation science and explicitly focusing on the pioneering colorectal ERAS protocol.

### Implementation Science Perspective

Implementation science is the scientific study of methods and strategies that help practitioners and policymakers incorporate evidence-based practices and research into their daily routines.^5^ This field aims to bridge the gap between what we know and what we do by systematically identifying and addressing the barriers that impede the implementation of proven health interventions and evidence-based practices. The branch plays a crucial role in integrating concepts from various disciplines, such as organizational behavior, clinical epidemiology, intervention science, health economics, adult education, and marketing.^6^ Additionally, healthcare researchers increasingly recognize the importance of implementation science. In the early 2000s, the United States established the quality enhancement research initiative (QUERI), and the United Kingdom established the National Institute for Health Research Service Delivery and Organization Program. These initiatives were created to promote the implementation of evidence-based practices and have been successful.

The lack of implementation of clinical advancements can be attributed to the rapid pace of modern biomedical research, which surpasses society’s ability to absorb them. This explains why some developments or changes take a long time to be accepted by the societies interested in them.^7^ The implementation process of ERAS protocols is likely relevant to the situation. ERAS protocols have been designed to meet the specific needs of medical fields and professionals within a required time frame, especially in countries with specific requirements for optimal perioperative care. However, in communities facing challenges beyond improving perioperative care, delays in implementing ERAS protocols are unavoidable. Interventions and evidence-based practices may not produce the expected outcome benefits if they are poorly implemented or not implemented at all. Even when effectively implemented, interventions and practice changes may still fail to deliver anticipated health benefits if their effectiveness is lost during implementation or if the intervention or practice was never effective in the first place.^8^

Several evidence-based initiatives with proven better patient outcomes, such as ERAS care bundles, have not been fully integrated into standard practice. The differences observed between the slow and rapid adoption of ERAS guidelines in clinical practice highlight the influential role of contextual factors in determining the speed and extent of their widespread use, in addition to their effectiveness. Ferlie et al.^9^ claimed that the presence of complex organizations containing many different professional groups hinders the spread of what should be implemented. This theory helps us explain barriers to the spread of an initiative in large, multiprofessional organizations in both healthcare and other settings, such as in ERAS programs. Surgeons, anesthesiologists, nurses, physiotherapists, and nutritionists are employed within unprofessional communities in which the same language is spoken and who have common internal learning processes. Additionally, social and cognitive boundaries between different professions may impede the spread as individual professionals work within unidisciplinary communities of practice.^9^ Hence, it is essential to address challenges like creating a unified and innovative language in a multidisciplinary working environment and aligning perspectives. This will facilitate the development of shared paths for the application of ERAS protocols.

The current evidence-based ERAS protocols face challenges in seamlessly integrating into clinical practice because of their scientific validity alone. The issue at hand is that each professional team aims to provide the highest level of evidence to patients. However, the reality is quite different. The effectiveness of ERAS protocols arises from the combined application of good clinical practice to the same patient. This can be understood by observing that patient outcomes improve as the compliance rate with the protocol increases.^10^ Implementation science should address this issue and provide a framework for this model.

More than just concise study results are necessary to ensure the regular adoption of a new clinical practice. The significance of training when integrating a new application into regular practice cannot be overstated. It is crucial that individuals who are expected to use this application receive proper training, are supervised, and receive feedback at the conclusion of training. However, a Cochrane meta-analysis showed that audit and feedback only increased target provider behaviors by 4.3%.^11^ Therefore, education and monitoring are not sufficient to change provider behaviors. The longstanding and persistent problem of healthcare providers not adopting effective clinical initiatives is influenced by factors beyond the initiative itself. External factors, such as a different professional society or nationality, also play a significant role in determining whether or not the initiatives are properly utilized.

The ERAS guidelines are far from being implemented under the current leadership. Protocols are often complex and multifaceted, with many interacting components. Items can be conceptualized as having “core components” (the essential and indispensable elements of the protocol) and “peripheral components” (adaptable elements, structures, and systems related to the intervention and organization into which it is being implemented).^8^ Due to its nature, a multifaceted strategy is needed to implement an ERAS protocol. It may include drawing baseline data, getting appropriate education regarding the whole process from a well-experienced group, establishing a registry of patients undergoing surgery using an ERAS protocol, and, last but not least, having both internal and external audits during and after completion of the implementation process. Therefore, the process should involve two main layers. The first layer identifies barriers to and facilitators of implementation across various levels of context, including patients, providers, organizations, and stakeholders such as policymakers. The next layer involves developing and implementing strategies to overcome these barriers and enhance facilitators to increase the adoption of evidence-based clinical initiatives.^12^

### Barriers and Potential Solutions

### • Healthcare System and Policy Level

ERAS is an evidence-based multidisciplinary perioperative care pathway. This concept of perioperative care brings together surgeons, anesthesiologists, nurses, physiotherapists, nutritionists, and even patients, all involved in the surgical care journey, to achieve favorable patient outcomes, as previously mentioned. This is best achieved by creating a multidisciplinary ERAS committee, which can be defined as a team that works well together and believes in the value of ERAS protocols.

In a hospital setting, the implementation of an ERAS program can be approached in two distinct ways from an organizational standpoint. The initial alternative can be executed by hospital management through top-down communication as a vertical structure. In this method, the hospital selects the individuals participating in the committee, regularly assesses the results, and devises corrective actions for any issues. Responsibility is assigned to one or a few individuals designated by hospital management. As a result, the support provided by other committee members may be limited, and the process may progress slowly. Nevertheless, the effectiveness of this approach lies in its strong enforcement ability. In the second method, the process progresses from bottom to top. This method involves team members forming an ERAS team and persuading hospital management to use data to emphasize increased service quality and patient satisfaction. In our country, the second option is more common to establish an ERAS program. However, it is easy to anticipate that the first option will become more prevalent in implementing ERAS protocols due to inevitable changes in national healthcare policies. For instance, the Ministry of Health has been developing a protocol for colorectal cancer, which has been in place nationwide since 2019 as part of a clinical quality improvement initiative.^13^ However, the current protocol does not adequately cover the perioperative care of surgical patients. In the future, integrating the elements of the evidence-based ERAS guideline into this national protocol could significantly accelerate the adoption of ERAS practices throughout the country. In order to accomplish this goal, it would be advisable for the ERAS Turkey Society and the Ministry of Health to carry out a collaborative study.

Imagine that the outcomes of patients following surgical procedures are regularly provided as feedback to both institutions and the public. In this case, such information will at least push institutions to produce higher-quality service, on average, toward the country’s average. It would even help to use a benchmarking approach that aims to achieve the “best in class”.^14^ Feedback on such information constitutes the most effective internal audit and regulation mechanism of the health system.^15^ Therefore, it might be possible to foresee that ERAS protocols will become much more popular due to the increased quality of health services, and institutions will be encouraged to implement ERAS protocols in all types of surgery with top-down instructions, as previously mentioned. In addition, improvement in performance reimbursements for centers implementing ERAS, according to the performance-based payment system itself, may also be encouraging for centers planning ERAS implementation.

A 2021 study in Turkey on the health services system multidimensional trust scale found that public trust in health services was at a medium level, indicating a lack of strong trust.^16^ Therefore, it is crucial to take measures to uphold public trust in healthcare systems when developing health policies. Becoming an ERAS center not only involves providing exceptional service but also building trust in the healthcare system using evidence-based data.

### • Organizational Level

Healthcare professionals in the ERAS program must collaborate fully, prioritizing patient care. Common perspective and good communication are the minimum requirements for this harmony. Therefore, the greatest challenge in the implementation process is the requirement of an interdisciplinary effort.^17^

Communication is a cultural phenomenon that should be developed to advance an ERAS program. However, given the time constraints, thoroughly discussing and making a mutual decision with each patient is often challenging. This is where the designated ERAS coordinator plays a critical role. The coordinator provides ERAS education to patients before surgery, monitors ERAS patients after surgery, collects patient data on adherence to the ERAS protocol, and evaluates patient outcomes. Additionally, an ERAS coordinator should collaborate closely with frontline care teams and serve as the communication channel between frontline care members and executive and leadership members throughout the process. The coordinator should also consistently collaborate with team members, such as the anesthesiologist, nutritionist, patients, and data collector. This collaboration includes sharing information and data, highlighting successes and areas for improvement, and updating care plans as necessary. Conducting regular structured meetings, either weekly or monthly, depending on the need, to discuss all ERAS cases and ensure compliance with ERAS items is crucial for success. The discussions and updates revolve around modifying order sets, documenting issues, flow sheet issues, and complications. In these meetings, ERAS champions from all different surgical specialties can be identified, and other team members and hospital management can reward them in various ways.

Institutions implementing their ERAS programs can initially have a smaller team. However, it is essential to ensure that all group members are fully committed. Guidelines are crucial for process management. Each institution can decide by considering its capabilities, goals, and what is essential for success. Thus, each ERAS committee can create revised guidelines specific to the procedure and the institute.

### • Patient Level

Different societies may have different attitudes toward health and disease. Supporting patients is a significant factor that influences barriers to and facilitators of care. In an ERAS program, patients play a major role in driving clinical care related to nutrition, mobilization, pain and symptom control, and hydration. Patients often desire to be actively involved in their care from diagnosis until recovery, although not always. In a study, patients who had face-to-face, semi-structured interviews with key stakeholders regarding their social support, satisfaction levels, preadmission information and education, pain management, and mobilization revealed that education level was considered an important barrier from the patient’s perspective.^18^ Therefore, the implementation process may involve various options regarding the level of education that can be delivered to the patient.

Patients may want to learn about ERAS and why it is important to follow the guidelines to support their surgical journey. Although patients may want to leave the hospital sooner, they are generally worried about the consequences after discharge. Patients who cannot advocate for themselves express interest in learning effective decision-making to advocate for themselves. The use of perioperative counseling and support, as well as yoga, meditation, mindfulness, and exercise, are potential strategies for managing stress. Almost 50% of patients should undergo colorectal surgery for cancer, and delays in test results and support for patients with earlier-stage cancers are barriers to treatment. Timely follow-up with the surgeon and postoperative communication with an ERAS coordinator can provide the patient with a sense of trust and well-being, along with valuable insight.

From the patient perspective, education strategies during the perioperative period might affect the implementation of ERAS. The mode of education (web-based, books, videos, face-to-face meetings) is a potential challenge. Strategies should be identified according to the features of the patients. Both patients and their families are involved in education planning. Options to support rural patients and address issues related to language, cognition, and elderly patients have been identified.

### Sustainability of the ERAS Program

A systematic implementation model is essential to guarantee the sustainability of ERAS programs. Although implementing change in a single service line is challenging, implementing system-wide changes requires extensive collaboration, change management, and optimization strategies. These strategies are necessary to ensure that the healthcare system seamlessly embraces these programs. The real challenge often begins after implementation: maintaining consistent standardization of care and compliance across departments. An implementation practice should not only propose “what works,” but also delve into what works where and why to make it sustainable. ERAS programs can be exciting at the start and initially successful, but they require ongoing commitment to process mapping, problem-solving, and compliance with ERAS protocols, which may diminish over time.^19^ To overcome such problems, hospital management can now appreciate and highlight the team and their successes. As is done in every quality process, the activities of the ERAS team can be conveyed to the entire hospital and perhaps even the university through advertising and promotional activities. An additional source of financial support can be created for the financial support that may be needed in the ERAS protocol, and additional financial support can even be provided to team members for their devoted efforts according to the rules of the performance system that already exists in the healthcare system.

### Real-World Implementations

To establish the best ERAS practice, a center generally requires three items: an ERAS evidence-based surgery-specific guideline, an ERAS Implementation Program (EIP) for change management, and an ERAS interactive audit system (EIAS). The EIAS is a web-based data entry and analysis system that tracks compliance with evidence-based guidelines established by the site-based ERAS team. The EIP includes an implementation program for change management, coaching, and supervision of an implementation team in training-the-trainer sessions, with a surgeon, an anesthesiologist, and a nurse leader acting as the coordinator for a specific type of surgery. The latter two are provided by Encare, which is in close collaboration with the ERAS society and enables continuous data-driven improvement of patient outcomes based on best practices and current research.

Many theories and frameworks of behavioral change exist. However, only a small number of them have been tested in robust research in healthcare settings. Alberta health services used the theoretical domains framework (TDF) within the QUERI model at individual and organizational levels to identify barriers to and facilitators of spreading and scaling the implementation process. The TDF aims to identify the primary aspects of centers and their requirements. This is a systematic method for moving from target behaviors to theoretical domains, behavior change techniques, and finally to a full implementation intervention. Psychological theories can be used within this framework to identify barriers to changing practices. In Alberta, the implementation of the ERAS was guided by the following questions and their answers: 1. Who needs to do what differently? 2. What barriers to and facilitators of change practice? 3. What strategies were used to address barriers and enablers? 4. What strategies were used to measure behavioral change and its impact on outcomes? As a result, the implementation had a positive impact on patient and healthcare system outcomes and was effectively applied across multiple institutions. The median overall guideline compliance was 39 in pre-ERAS and 60% in post-ERAS patients. The median length of stay was six days for pre-ERAS and 4.5 days for post-ERAS patients. In addition, complications and readmission rates were reduced.^20^

Although utilizing the three items may seem sufficient for practical implementation, the costs associated with EIAS and EIP are significant, particularly in low- and middle-income countries. There are yet to be registered ERAS centers and ERAS centers of excellence from Turkey in the ERAS Society due to budget shortages. In many cases, determining costs accurately can be challenging, and the availability of resources may affect implementation more directly.

Finally, future ERAS strategies should shift away from the endpoints of early recovery and shortened length of stay and focus more on discharge problems, such as the risk of thromboembolic complications, postoperative orthostatic intolerance, late cognitive dysfunction, muscle function, and postoperative sleep disturbances.^21^ Therefore, the new implementation strategies should focus on emerging trends to ensure the incorporation of more effective results into the guidelines.

## Conclusion

As noted by famous Romanian sculptor, painter, and photographer Constantin Brancusi, “Seeing far is one thing, going there is another”. ERAS protocols may have failed to be implemented for various reasons, although they are very efficient. Barriers to implementation may arise at the policy, organizational, provider, and patient levels. In addition to theory, in practice, the success of an ERAS implementation relies on motivated clinicians working together to engage stakeholders, understand workflow processes, and overcome barriers to the delivery of evidence-based care. We look forward to witnessing progress in the years to come and reaching a point where there is seamless integration of research into practice and policy.
